# Comparison of Ray Tracing and Monte Carlo Calculation Algorithms for Spine Lesions Treated With CyberKnife

**DOI:** 10.3389/fonc.2022.898175

**Published:** 2022-05-04

**Authors:** Jun Li, Xile Zhang, Yuxi Pan, Hongqing Zhuang, Ruijie Yang

**Affiliations:** Department of Radiation Oncology, Peking University Third Hospital, Beijing, China

**Keywords:** CyberKnife, Ray tracing, Monte Carlo, stereotactic body radiation therapy, spine tumor

## Abstract

**Purpose:**

This study attempts to evaluate Ray Tracing (RT) and Monte Carlo (MC) algorithms for CyberKnife treatments of spine lesions and determine whether the MC algorithm is necessary for all spine treatment and compare the RT algorithm and MC algorithm at various spine lesion sites.

**Methods:**

The CyberKnife is used for stereotactic body radiotherapy for lesions in the cervical spine (30), thoracic spine (50), lumbar spine (30), and sacral spine (15). Dose was calculated using RT and MC algorithms for patients planned with the same beam angles and monitor units. Dose-volume histograms of the target and selected critical structures are evaluated.

**Results:**

The average target coverage of prescribed dose with MC algorithms was 94.80%, 88.47%, 92.52%, and 93.41%, respectively, in cervical, thoracic, lumbar, and sacral spine. For the thoracic spine, the RT algorithm significantly overestimates the percentage volume of the target covered by the prescribed dose, as well as overestimates doses to organs at risk in most cases, including lung, spinal cord, and esophagus. For cervical, lumbar, and sacral spine, the differences of the target coverage of prescription dose were generally less than 3% between the RT and MC algorithms. The differences of doses to organs at risk varied with lesion sites and surrounding organs.

**Conclusions:**

In the thoracic spine lesions with beams through air cavities, RT algorithm should be limited and verified with MC algorithm, but the RT algorithm is adequate for treatment of cervical, lumbar, and sacral spine lesions without or with a small amount of beams passing through the lungs.

## Introduction

Stereotactic body radiation therapy (SBRT) technology has the potential to increase the dose of the tumor and reduce the dose to normal tissue, so it can obtain higher tumor control probability and lower normal tissue complication probability (1). As the typical equipment for SBRT technology, CyberKnife (CK) has been increasingly used for spinal lesion treatment in modern radiotherapy. However, non-isocentric, non-coplanar beams of SBRT based on CK are more complicated than conventional radiotherapy. Thus, the accuracy of dose calculation is crucial for effective treatment.

Two types of dose calculation algorithms, Ray Tracing (RT) and Monte Carlo (MC), are used for CK. Many studies have reported that the dose calculated by the RT and MC algorithms were significantly different in heterogeneous tissues. The degree of difference is influenced by many factors, including tumor size, location, peripheral tissue characteristics, and collimator aperture (2–5). Compared to the RT algorithm, the MC algorithm is able to take into consideration the tissue heterogeneities, while density scaling functions and effective depth correction factors are not required (6, 7). Therefore, the MC algorithm for CK could provide more accurate dose distribution calculations in regions of lateral electron disequilibrium. However, dose optimization of the MC algorithm is computationally cumbersome, requiring more time to carry out, particularly with lower uncertainty levels. Compared with the RT algorithm, the MC algorithm needs more computing power and personnel time (8, 9). Given the current state of technology and computing power, there exists a limitation to the clinical implementation of quality MC‐calculated treatment plans. Moreover, some studies have shown that the dosimetric difference between RT and MC may not be appreciably significant, such as treatment sites with quasi-homogeneous tissues (10).

In addition, due to the specific geometry of the CK layout, the space below the treatment couch plane cannot be accessed by CK. The posterior radiation beams for patients are prohibited in the supine position. Thus beams aimed at posterior spinal lesions had to traverse a substantial length of normal tissues on their way to the target. As we know, the spine is composed of cervical vertebrae, thoracic vertebrae, lumbar vertebrae, and sacrum bone. There are various adjacent tissues close to different types of vertebrae. These tissues include homogeneous tissues, such as the spinal cord, the kidneys and heterogeneous tissues such as the lungs, the esophagus, the intestinal tract and so on. Therefore, the relationship between the spine and its neighboring tissues is quite complex. For spine lesions, it is unknown whether it is necessary for all lesions of the spine using the MC algorithm to improve the dose calculation accuracy or whether the treatment plan with the RT algorithm could be acceptable in clinical treatment. Due to the lack of data on the choice of the algorithm in previous studies, this question needs further exploration. In our study, dose distributions calculated by MC and RT are compared for patients with cervical, thoracic, lumbar, and sacral spine lesions, and to determine whether CK based SBRT using RT algorithm is comparable dosimetrically to that of MC for spinal lesions.

## Methods and Materials

### Treatment Planning and Dose Prescription

This is a retrospective study on spinal tumor patients treated with SBRT by CyberKnife VSI (Accuray Inc., Sunnyvale, CA) from 2018 to 2020. The study was approved by the institutional review board of our hospital. One hundred twenty-five spinal lesions were treated, 30 for the cervical spine, 50 for the thoracic spine, 30 for the lumbar spine, and 15 for the sacral spine.

Patients were simulated in the supine position. CT scan was acquired with a slice thickness of 1.5 mm and used for structures delineation and dose calculation. The first and second cervical spinal tumors of treatment plans were designed using the 6D-Skull tracking method, other sites using the spine tracking method. The dose was calculated using the RT algorithm in the CyberKnife TPS MultiPlan v.4.6 (Accuray Inc.). Treatment plans were generated using 1-4 IRIS aperture collimators and an average 148 (range 104-227) non-coplanar non-isocentric beams. The smallest collimator was 10 mm in diameter and the largest collimator was 60 mm in diameter. The size of dose calculation grid x and y is less than 1mm, z is 1.5mm. Characteristics of the patient and associated plan parameters are shown in **Table 1**. The PTV volume ranged from 51.7 cc to 319.8cc. Patients were treated in 3 fractions with a dose of 24Gy-30Gy. The PTV dose was prescribed at 68%-84% isodose level. The dose coverage of PTV ranged from 93.9% to 96.8%.

**Table 1:** Patient and tumor characteristics.

Site	Number of patients	Tumor location	Prescription dose (Gy)	Number of fractions	PrescriptionIsodose	CollimatorSizes (mm)	PTV Volume (cc)
Cervical spine	9	C1-2	27-30	3	70%-77%	10-50	87.2-204.6
	14	C3-5	24-30				
	7	C6-7	27-30				
Thoracic spine	14	T1-3	27-30	3	68%-80%	12.5-50	51.7-303.5
	11	T4-6	24-30				
	10	T7-9	27-30				
	15	T10-12	24-30				
Lumbar spine	13	L1-2	24-30	3	69%-81%	10-60	108.3-319.8
	17	L3-5	24-30				
Sacral spine	15	S1	27-30	3	71%-84%	12.5-60	67.4-228.1

### Plans Recalculated Using MC Algorithm

Based on the plans computed using the RT algorithm introduced above, plans were recalculated using the MC algorithm without re-optimization. For the MC calculation, the collimator size, the number of beams, and the monitor units were kept unchanged from the original RT plans. The prescription for each plan was not renormalized. MC plans were calculated using high-resolution mode with a clinically meaningful uncertainty level of 1.5%.

### Dosimetric Comparison Between Ray Tracing and Monte Carlo Algorithms

Dose calculations, using the MC and RT algorithms, were compared by analyzing the dose-volume histograms (DVHs) for the plan target volume (PTV) and organs at risk (OARs). PTV was assessed by V_100_. For the OARs, V_5_ and mean dose were used for the lung. The maximum dose, D_0.1cc_, D_1cc_ and D_5cc_ were used for the esophagus, spinal cord, and bowels. Mean dose and V_16_ were used for the kidneys. Comparison between the dosimetric parameters generated by the RT and MC algorithms were analyzed with a t-test. P-value below 0.01 indicates statistically significant differences between mean values of the data sets.

## Results

### Dosimetric Comparisons Between RT and MC for Cervical Spine

**Figure 1:**
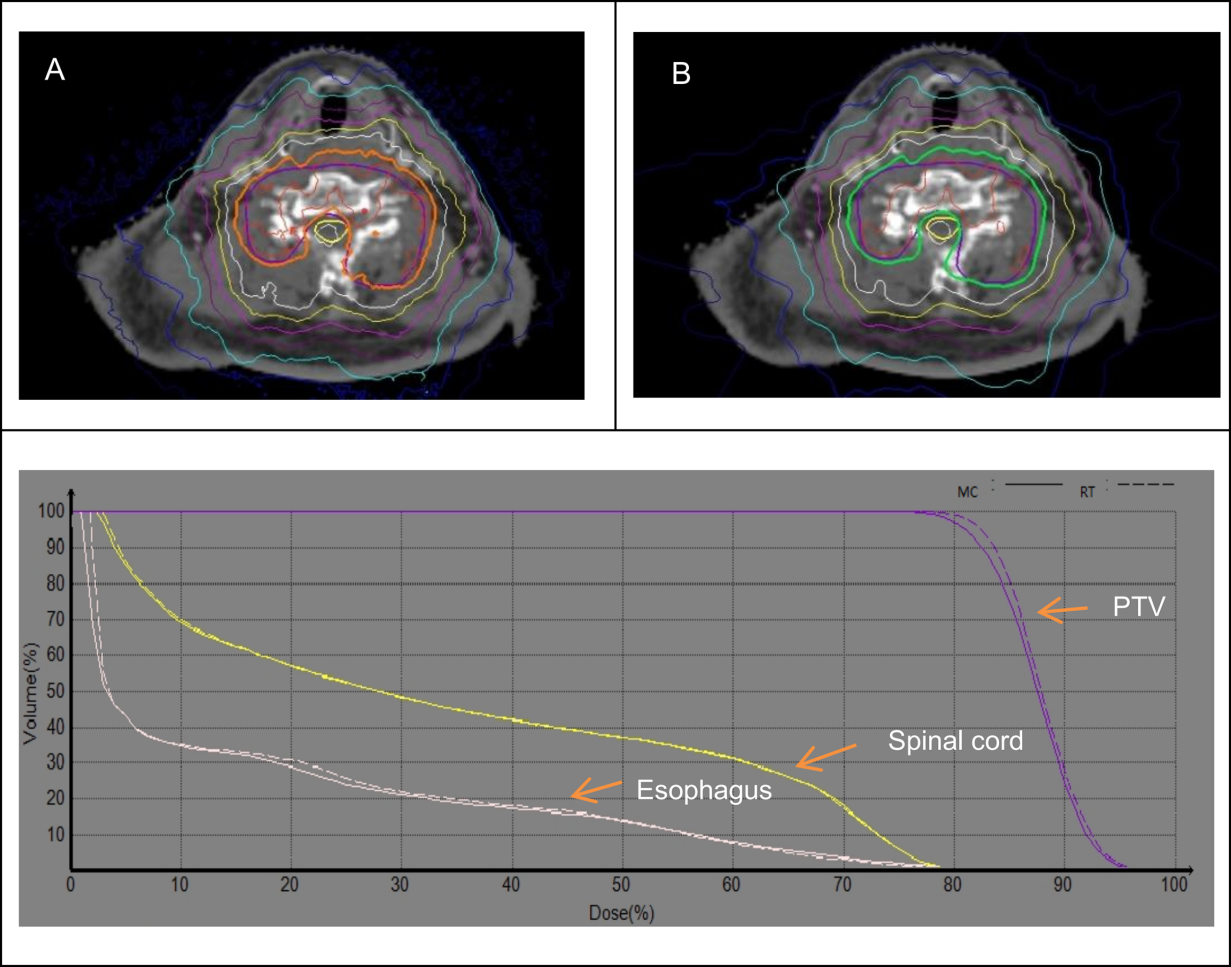
Dose distribution and DVH between the MC **(A)** and RT **(B)** algorithms for cervical spine.

**Figure 1** demonstrates the dose distribution and DVHs for cervical spine lesions between the RT and MC algorithms. There was no significant difference for target and OARs. Compared with the region of high dose, the change of the low dose region is relatively obvious. In order to quantify specifically the effect on the dose differences using the RT and MC algorithms, the dosimetric parameters were given in **Table 2**. For C1-2, compared to MC plans, the V_100_ of PTV is on average 0.88% lower in RT plans, and the average RT/MC ratio of PTV V_100_ was 0.990, ranged from 0.984 to 1.012. For C3-7, compared to MC plans, the V_100_ of PTV was on average 1.41% higher in RT plans. The Average RT/MC ratio of PTV V_100_ was 1.015, ranged from 0.989 to 1.021. For OARs, there was no significant discrepancy between RT and MC algorithms. RT/MC ratio ranged from 0.978 to 1.023 for dosimetric parameters of the spinal cord and esophagus.

**Table 2:** Dose parameters of cervical spine lesions.

Site	Dosimetric parameter	Ray Tracing(mean ± SD)	Monte Carlo(mean ± SD)	P-Value
C1-2	PTV			
	V_100%_	95.40% ± 0.43%	96.28% ± 1.41%	<0.01
	Spinal Cord			
	D_max_ (Gy)	24.15 ± 3.94	24.48 ± 3.83	0.12
	D_0.1cc_ (Gy)	20.66 ± 3.98	21.09 ± 3.84	0.08
	D_1cc_ (Gy)	15.85 ± 4.79	16.13 ± 4.74	0.15
	D_5cc_ (Gy)	10.23 ± 6.77	10.40 ± 6.33	0.46
	Oral mucosa			
	D_max_ (Gy)	24.86 ± 67.3	25.00 ± 6.92	0.17
	D_0.1cc_ (Gy)	23.20 ± 6.60	23.31 ± 6.72	0.51
	D_1cc_ (Gy)	20.14 ± 6.25	20.27 ± 6.47	0.33
	D_5cc_ (Gy)	15.85 ± 5.38	15.93 ± 5.69	0.42
C3-7	PTV			
	V_100_	95.33% ± 0.50%	93.92% ± 1.27%	<0.01
	Spinal Cord			
	D_max_ (Gy)	25.15 ± 2.71	24.74 ± 2.58	0.02
	D_0.1cc_ (Gy)	22.42 ± 3.24	22.15 ± 3.06	0.08
	D_1cc_ (Gy)	17.53 ± 4.82	16.97 ± 4.52	0.05
	D_5cc_ (Gy)	8.65 ± 6.52	8.24 ± 6.53	0.42
	Esophagus			
	Dmax	27.04 ± 3.48	26.69 ± 3.12	0.07
	D_0.1cc_ (Gy)	24.81 ± 4.74	24.34 ± 4.26	0.21
	D_1cc_ (Gy)	20.72 ± 4.96	20.34 ± 4.58	0.13
	D_5cc_ (Gy)	13.56 ± 5.18	13.24 ± 4.70	0.07

### Dosimetric Comparisons Between RT and MC Algorithms for Thoracic Spine

**Table 3** shows the results using RT and MC algorithms for target and OARs in the thoracic spine. The dose discrepancy in T1-3 and T10-12 was smaller than in T4-9 between the RT and MC algorithms. For T1-3 and T10-12, compared to MC plans, V_100_ of PTV was on average 3.07% and 3.58% lower in RT plans. In T1-3, the average RT/MC ratio of PTV V_100_ was 1.034, range from 1.017 to 1.042. In T10-12, the average RT/MC ratio of PTV V_100_ was 1.039, range from 1.026 to 1.048. For these two sites, there was no significant dosimetric discrepancy in the esophagus and spinal cord, and RT/MC ratio ranged from 0.976 to 1.030. The average deviation in V_5_ of lung between MC and RT algorithms were generally less than 3%, and the average RT/MC ratio of D_mean_ was 1.038, ranged from 0.984 to 1.048.

**Table 3:** Dose parameters of thoracic lesions.

Site	Dosimetric parameter	Ray Tracing(mean ± SD)	Monte Carlo(mean ± SD)	P-Value
T1-3	PTV			
	V_100%_	94.93% ± 0.39%	91.86% ± 3.46%	<0.01
	Spinal Cord			
	D_max_ (Gy)	22.01 ± 1.88	21.59 ± 2.01	0.02
	D_0.1cc_ (Gy)	19.44 ± 3.22	18.90 ± 2.42	0.03
	D_1cc_ (Gy)	15.85 ± 1.79	15.46 ± 2.00	0.33
	D_5cc_ (Gy)	10.23 ± 3.49	10.11 ± 3.15	0.46
	Esophagus			
	D_max_ (Gy)	25.10 ± 2.23	24.36 ± 2.03	0.02
	D_0.1cc_ (Gy)	22.07 ± 1.47	21.25 ± 2.06	0.02
	D_1cc_ (Gy)	19.20 ± 1.74	18.51 ± 1.62	0.02
	D_5cc_ (Gy)	13.69 ± 2.51	13.02 ± 2.14	0.42
	Left Lung			
	V_5_	20.2% ± 4.6%	19.1% ± 8.4%	0.02
	D_mean_ (Gy)	3.21 ± 0.48	3.15.3 ± 0.71	0.04
	Right Lung			
	V_5_	26.8% ± 6.7%	24.3% ± 9.3%	0.02
	D_mean_ (Gy)	4.75 ± 1.06	4.67 ± 1.23	0.02
T4-9	PTV			
	V_100%_	95.22% ± 0.51%	82.67% ± 13.68%	<0.01
	Spinal Cord			
	D_max_	22.80 ± 3.93	21.76 ± 4.24	<0.01
	D_0.1cc_ (Gy)	19.28 ± 3.81	18.32 ± 3.57	<0.01
	D_1cc_ (Gy)	16.82 ± 4.45	16.47 ± 4.02	0.05
	D_5cc_ (Gy)	7.49 ± 5.06	7.22 ± 5.61	0.42
	Esophagus			
	D_max_ (Gy)	23.57 ± 3.52	22.48 ± 2.84	<0.01
	D_0.1cc_ (Gy)	21.22 ± 3.27	20.16 ± 3.53	<0.01
	D_1cc_ (Gy)	18.46 ± 3.83	17.94 ± 3.53	0.03
	D_5cc_ (Gy)	13.86 ± 4.35	13.34 ± 4.67	0.02
	Left Lung			
	V_5_	28.40% ± 18.4%	25.97 ± 13.80%	<0.01
	D_mean_ (Gy)	5.77 ± 1.83	5.29 ± 1.55	<0.01
	Right Lung			
	V_5_	34.2% ± 18.9%	31.8% ± 15.2%	<0.01
	D_mean_ (Gy)	6.53 ± 2.25	6.01 ± 1.72	<0.01
T10-12	PTV			
	V_100%_	95.14% ± 0.27%	91.56% ± 2.18%	<0.01
	Spinal Cord			
	D_max_	22.61 ± 2.80	22.09 ± 2.89	0.02
	D_0.1cc_ (Gy)	20.71 ± 2.39	20.14 ± 2.52	0.05
	D_1cc_ (Gy)	18.01 ± 241	17.57 ± 2.45	0.03
	D_5cc_ (Gy)	94.2 ± 6.74	9.13 ± 7.20	0.07
	Esophagus			
	D_max_ (Gy)	20.71 ± 3.78	20.28 ± 4.14	0.07
	D_0.1cc_ (Gy)	18.83 ± 4.15	18.60 ± 4.14	0.21
	D_1cc_ (Gy)	16.35 ± 4.74	15.93 ± 5.01	0.01
	D_5cc_ (Gy)	12.75 ± 5.76	12.31 ± 5.95	0.01
	Left Lung			
	V_5_	21.11% ± 14.8%	19.81% ± 13.90%	0.02
	D_mean_ (Gy)	4.36 ± 2.42	4.21 ± 1.61	0.03
	Right Lung			
	V_5_	15.22% ± 8.6%	14.72% ± 10.7%	0.02
	D_mean_ (Gy)	3.66 ± 1.56	3.53 ± 1.34	0.03

**Figure 2:**
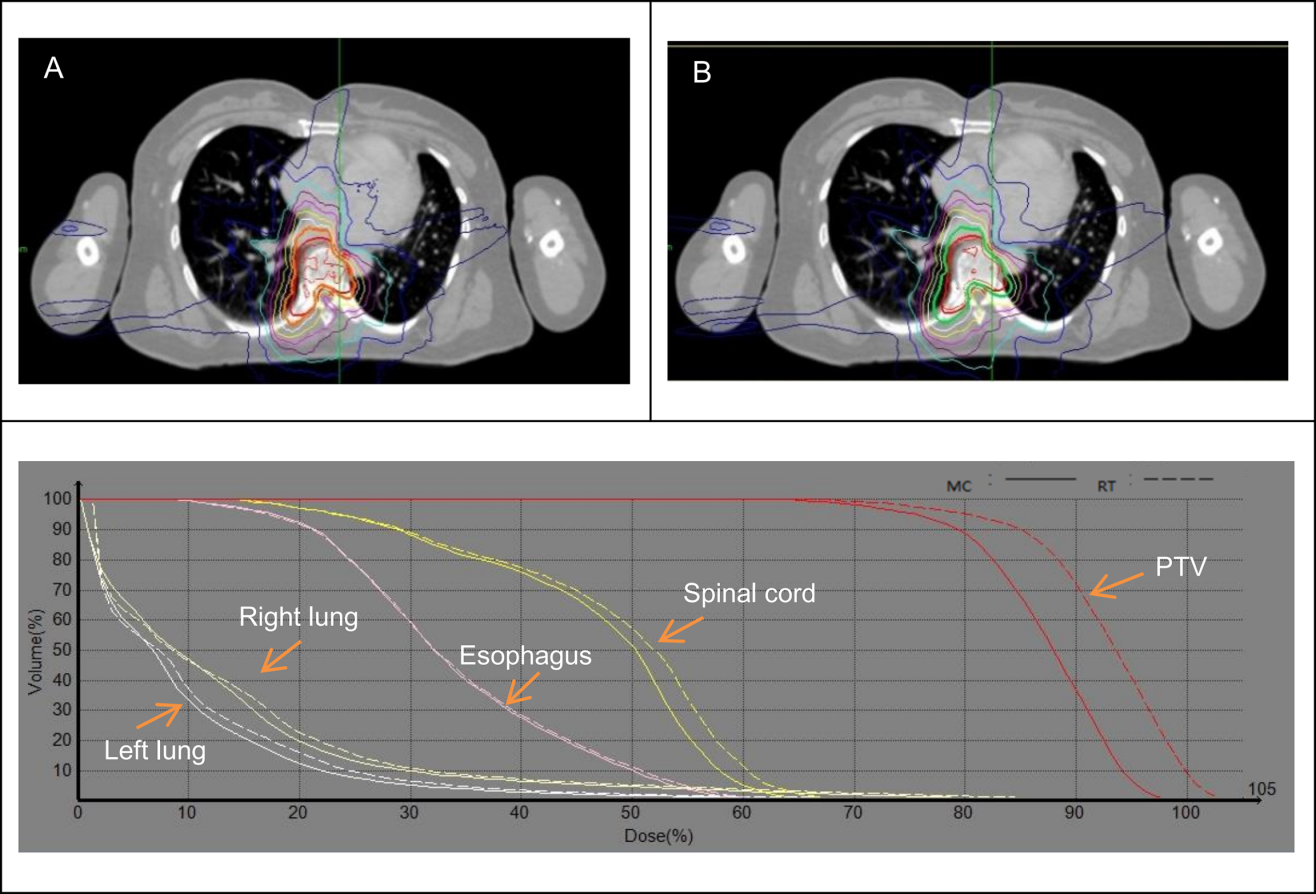
Dose distribution and DVH between the MC **(A)** and RT **(B)** algorithms for 6th thoracic spine.

For T4-9, the average V_100_ of PTV was 12.55% lower in MC plans than RT plans, the average RT/MC ratio of PTV V_100_ was 1.15, range from 1.066 to 1.381. Maximum dose and D_0.1cc_ for spinal cord and esophagus were statistically different (P<0.01) between RT and MC algorithms. RT/MC ratio of the spinal cord in the maximum dose and D_0.1cc_ ranged from 0.942 to 1.092, and 0.966 to 1.089, respectively. RT/MC ratio of the esophagus in the maximum dose and D_0.1cc_ ranged from 0.957 to 1.078, and 0.982 to 1.058, respectively. Meanwhile D_mean_ and V_5_ for lungs were statistically different (P<0.01). The average deviations were generally less than 5% in V_5_ and less than 10% in D_mean_. Based on the results shown in **Figure 2**, we found that the RT algorithm obviously overestimated the V_100_ of PTV compared to the MC algorithm. Moreover, at the lung-bone interface, the isodose line tends to pull toward the spine, and the target closest to the lung will have the highest dose falloff. Therefore, the minimum dose of target decreased observably at the lung-bone interface. In addition, the RT algorithm slightly overestimated the dose to lung compared to the MC algorithm. However, for other OARs farther away from the target (low dose region of OARs), the differences between MC and RT algorithms were almost negligible.

### Dosimetric Comparisons Between RT and MC Algorithms for Lumbar Spine

**Figure 3:**
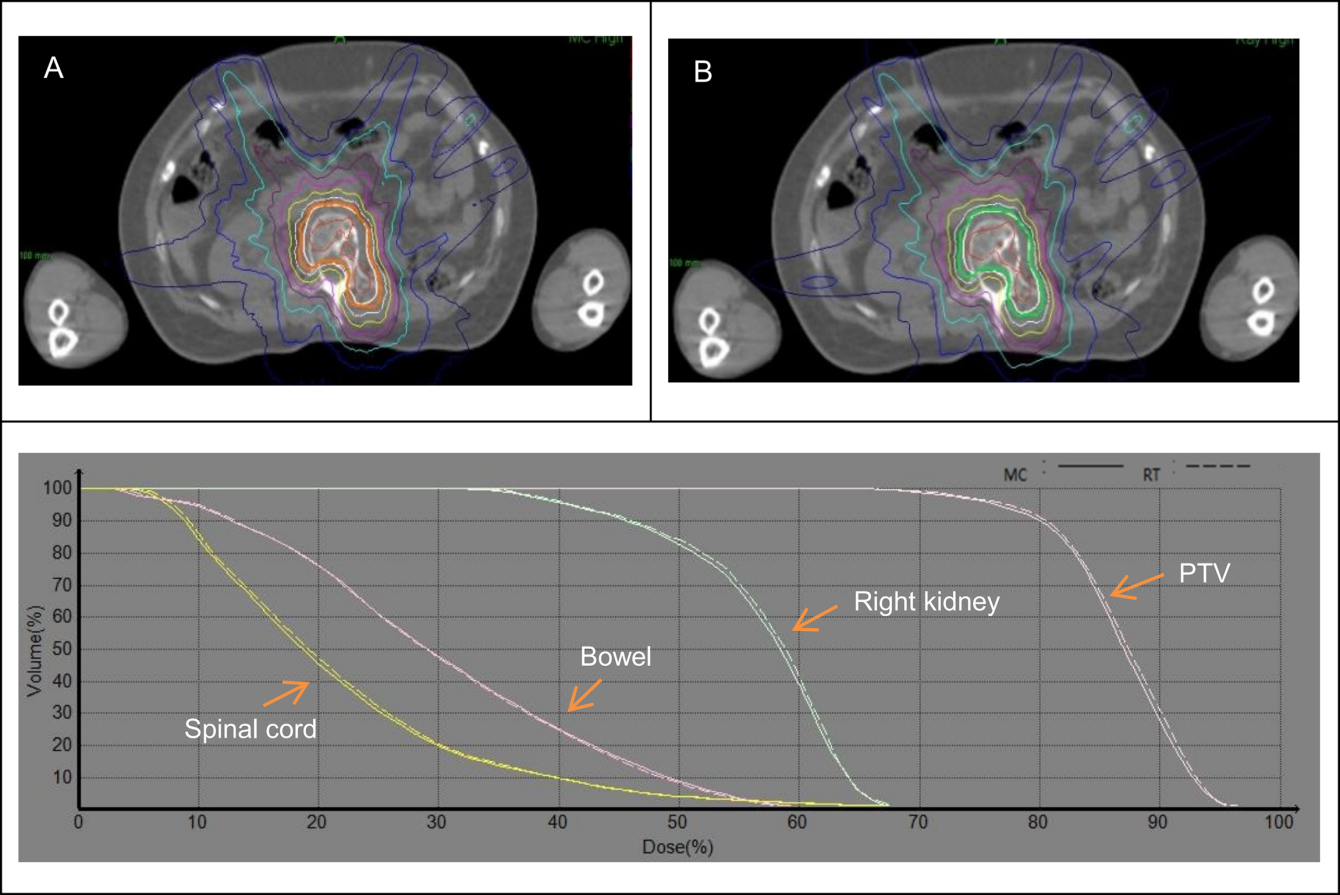
Dose distribution and DVH between the MC **(A)** and RT **(B)** algorithms for lumbar spine.

**Table 4** shows the comparison of dosimetric parameters between RT and MC algorithms for target and OARs in the lumbar spine. The average V_100_ of PTV was 2.44% greater in MC plans than RT plans, and the average RT/MC ratio was 1.026, and ranged from 1.008 to 1.032. We found it was still inevitable that a small number of beams would penetrate the lung tissue in L1 or L2 plans. So target coverage of prescription dose would be lower than other lumbar spines. For OARs, the dose difference between the RT and MC algorithms was not remarkable, and RT/MC ratio ranged from 0.982 to 1.028. From DVHs shown in **Figure 3**, the coverage of PTV and dose of OARs was basically unchanged.

**Table 4:** Dose parameters of lumbar lesions.

Site	Dosimetric parameter	Ray Tracing(mean ± SD)	Monte Carlo(mean ± SD)	p-Value
L1-5	PTV			
	V_100%_	95.3% ± 0.58%	92.86% ± 1.14%	<0.01
	Spinal Cord/ Cauda equina			
	D_max_	25.80 ± 4.35	25.54 ± 4.46	0.02
	D_0.1cc_ (Gy)	23.83 ± 4.60	23.54 ± 4.97	0.08
	D_1cc_ (Gy)	20.71 ± 4.14	20.49 ± 4.50	0.15
	D_5cc_ (Gy)	12.60 ± 4.49	12.46 ± 4.95	0.46
	Bowel			
	Dmax	23.28 ± 3.14	22.93 ± 2.35	0.17
	D_0.1_ (Gy)	22.01 ± 3.86	21.89 ± 3.09	0.51
	D_1cc_ (Gy)	20.21 ± 4.06	20.55 ± 6.48	0.33
	D_5cc_ (Gy)	16.69 ± 4.04	16.96 ± 4.86	0.04
	Stomach			
	Dmax	16.72 ± 7.34	16.62 ± 7.18	0.04
	D_0.1_ (Gy)	14.64 ± 6.89	14.51 ± 6.52	0.45
	D_1cc_ (Gy)	12.40 ± 7.03	12.18 ± 7.18	0.06
	D_5cc_ (Gy)	10.13 ± 6.55	9.78 ± 6.73	0.02
	Left Kidney			
	D_mean_(Gy)	10.24 ± 3.48	10.59 ± 3.12	0.07
	V_1600_ (cc)	81.30 ± 4.40	79.7 ± 6.20	0.21
	Right Kidney			
	D_mean_ (Gy)	12.28 ± 4.74	12.14 ± 4.26	0.11
	V_1600_ (cc)	10.43 ± 4.96	10.29 ± 4.58	0.13

### Dosimetric Comparisons Between RT and MC Algorithms for Sacral Spine

**Figure 4:**
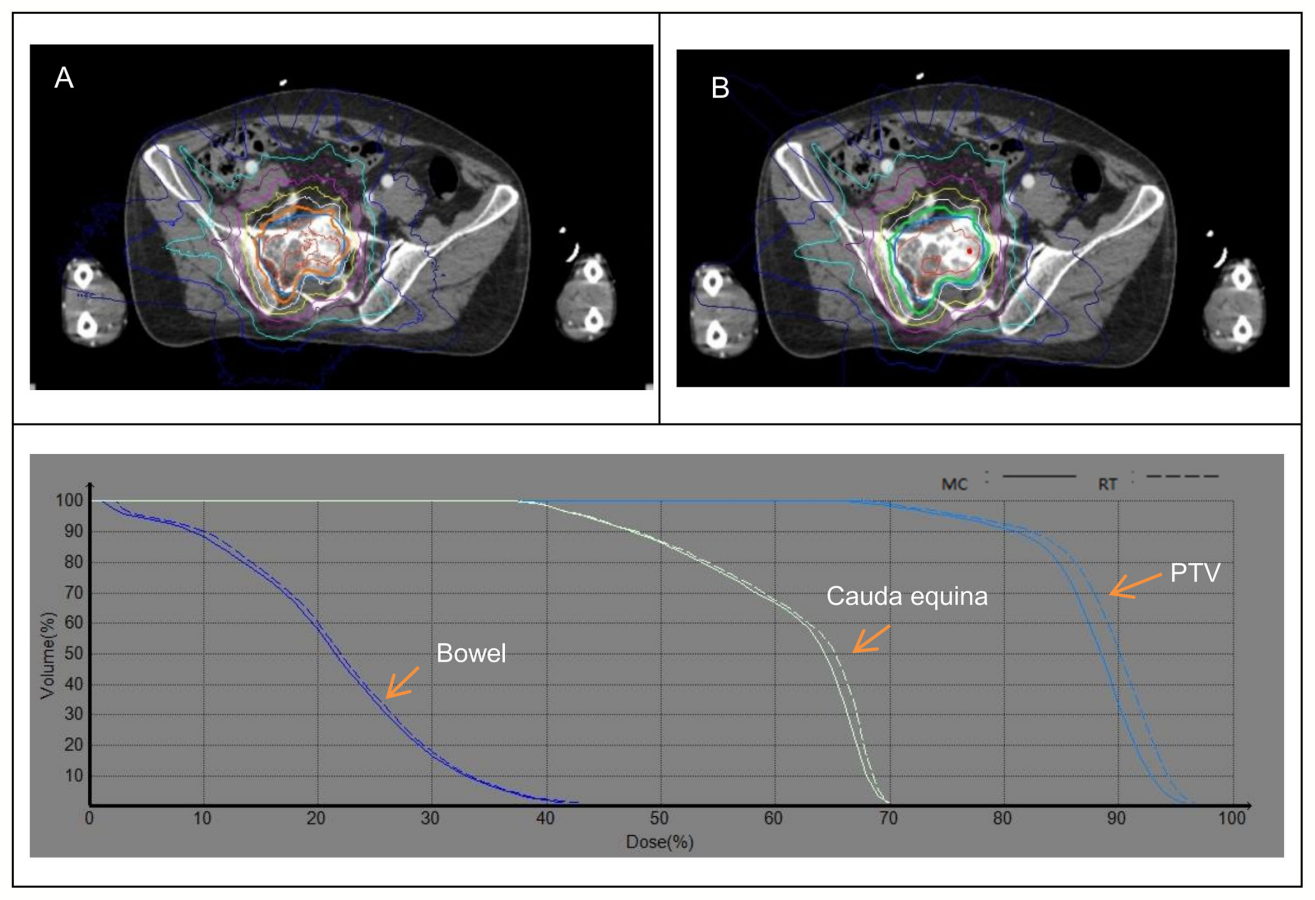
Dose distribution and DVH between the MC **(A)** and RT **(B)** algorithms for sacral spine.

The comparison of the dosimetric parameter between RT and MC calculations for target and OARs of the sacral spine is shown in **Table 5**. The average PTV coverage of prescription dose calculated using RT algorithm was uniformly better than the average PTV coverage using MC by up to 1.36%, and RT/MC ratio ranged from 1.003 to 1.019. For OARs, the dose deviation between the RT and MC algorithms was not remarkable and RT/MC ratio ranged from 0.982 to 1.028. As shown in **Figure 4**, the increased dose was displayed in RT plans compared to MC plans in DVHs. But the dose distribution of the MC algorithm was closer to the RT algorithm for OARs and PTV coverage of prescription dose.

**Table 5:** Dose parameters of sacral lesions.

Site	Dosimetric parameter	Ray Tracing (mean ± SD)	Monte Carlo (mean ± SD)	p-Value
S1	PTV			
	V_100%_	95.23% ± 0.67%	93.87% ± 1.02%	<0.01
	Cauda equina			
	D_max_	23.51 ± 3.44	23.38 ± 3.24	0.02
	D_0.1cc_	20.80 ± 3.69	20.90 ± 3.54	0.02
	D_1cc_	15.85 ± 4.03	15.73 ± 3.88	0.15
	D_5cc_	12.13 ± 3.77	12.22 ± 3.34	0.46
	Bowel			
	D_max_	24.91 ± 5.64	24.70 ± 5.42	0.17
	D_0.1_	23.01 ± 5.60	23.15 ± 5.47	0.32
	D_1cc_	20.34 ± 5.25	20.25 ± 5.18	0.51
	D_5cc_	15.85 ± 5.46	15.54 ± 5.33	0.04

## Discussions

CK is a widely used modality to treat spinal tumors due to its highly conformal dose distributions, steep gradient, and near real‐time image‐guidance system (11–13). The CK-based SBRT appears to be safe and effective at tumor control and symptom management in spinal patients (14, 15).

Meanwhile, CK spinal radiosurgery suffers from a lack of posterior beams due to mechanic design limitations, so that many beams pass through other tissues before reaching the spine lesions. Thus, the dose-relationship between spinal lesions of different sites and their adjacent organs is extremely complicated. Tissue heterogeneity correction will have a major effect on dose distribution. However, at present, dose calculation methods have not been strictly specified in clinical trials of spine SBRT. Although MC-based methods are increasingly recognized as the most appropriate, they have an uncertainty associated with the calculation results which needs to be added significantly to the treatment planning time (16, 17). Therefore, not all sites will benefit from the MC algorithm.

How to balance the time efficiency, calculation accuracy, and strategy of algorithm selection is the key. Our institutional datasets with spine SBRT were reviewed to determine the impact of RT versus MC algorithm on target coverage and dose exposure to the OARs. For the cervical spine, the coverage of the target using MC algorithm was slightly increased compared to the RT algorithm for some plans of C1 and/or C2. For the C3-C7, lumbar spine, and sacral spine, the RT algorithm commonly decreased the coverage of targets in small amounts. No significant dose differences were found for the OARs between the two algorithms. The deviation in the dose of OARs and coverage of target resulted in a maximum of 3.2% when comparing the RT algorithm with the MC algorithm. These results were similar to the previous study that showed the calculation algorithms RT and MC were equivalent in relatively homogeneous regions with dose deviation within 4% (10). The discrepancy in these values would not have been a determining factor as to which algorithm to utilize in treatment. Thus, the use of the RT algorithm in cervical, lumbar, and sacral spine lesions is to be regarded as sufficient and appropriate.

However, this situation was markedly changed in the heterogeneous tissue with MC calculation. For the thoracic spine, a significant difference in the dose distribution between the RT and MC algorithm is detected. Especially, in the middle thoracic spine (T6-T9), the MC algorithm resulted in an average loss in the coverage of PTV of 12.55%, and up to a maximum loss of 26.19%, consistent with the previous report (9). It prompted that the change in dose distribution due to the tissue heterogeneity effect was greater in lower-density structures. In the assumption that MC was the algorithm of highest accuracy it was shown that RT vastly overestimated the target coverage in the middle thoracic spine. With regard to the OARs, the maximum dose discrepancy could be up to 9.2%. Although the average of the dosimetric parameters mainly decreased in the MC plans, some results showed an increase. This means that the RT algorithm probably underestimates the exposure to OARs. For the spinal cord and esophagus, the dose increase could be up to 5.8% and 4.3%, respectively. This could result in a significant impact on the potential for cord and esophagus injury, especially in the spinal re-irradiation setting where treatment doses and OARs constraints are pushed to tolerance levels. Conversely, the decreased doses resulting from the MC recalculation were also consistently observed in the lungs. The average differences in V_5_ and D_mean_ between the RT and MC plans amounted to 5% and 10%, respectively. It was similar to report evaluating calculation methods in lung cancer (7). Although the overestimation of the lung dose may not lead to any significant treatment-related morbidity, it may reduce the effective tumor dose one can provide to at-risk tumor volumes, taking into account normal tissue tolerances (9).

By contrast, in the upper (T1-T3) and lower thoracic spine (T10-T12), the dose differences were much smaller than in the middle thoracic spine (T4-T9) for target and OARs. The maximum difference of target coverage was less than 5%. Meanwhile, for the spinal cord and esophagus, the maximum differences were both less than 3%. Compared with the MC algorithm, the dose to the lungs was not consistently overestimated with the RT algorithm, but there was no significant difference. Because these two sites had less nonadjacent tissue, and only a small number of beams pass through the lung tissues, resulting in slight changes in RT plans compared to MC plans. Thus, the RT algorithm perhaps could be utilized in the upper and lower thoracic spines. The target coverage might be improved by minimizing or avoiding beams traversing the lungs during the planning optimization. There had been evidence that the discrepancies of dose could be effectively reduced in this way (17). Additionally, the use of larger size collimators and lower isodose lines were expected to improve the dosimetric accuracy of RT computation (3, 18). Although this way was not a standard, it was a practical approach to achieve prescription goals.

A limitation of the present study was the lack of an assessment of the impact of metal implants on dose calculation accuracy. Some patients with multiple vertebral metastases who underwent spine SBRT had metal implants inserted for fixation of the vertebral bodies. It is important to evaluate the effects of these metal implants on spine SBRT, as investigated by some researchers (19, 20). In addition, plans were not subsequently optimized based on the MC algorithm. Therefore, the extent to which this algorithm can be used to improve the dosimetric parameters is unclear (10).

## Conclusion

For cervical, lumbar, and sacral spine lesions, the RT algorithm can be regarded as sufficient and appropriate for accurate calculation of dose. Besides, for the upper and lower thoracic spine, the RT algorithm might also be applied by reducing the number of the beams passing through the lungs. However, in the middle thoracic spine, the RT algorithm should be limited and always verified with the MC algorithm.

## Data Availability Statement

The original contributions presented in the study are included in the article/supplementary material. Further inquiries can be directed to the corresponding author.

## Author Contributions

JL conceived and designed the study. JL, XZ, and YP performed the experiments. JL wrote the paper. RY and HZ reviewed and edited the manuscript. All authors read and approved the manuscript.

## Funding

This work was supported by the National Key Research and Development Program (No. 2020YFE0202500), Beijing Municipal Commission of Science and Technology Collaborative Innovation Project (Z201100005620012), Capital’s Funds for Health Improvement and Research (2020-2Z-40919), and China International Medical Foundation (HDRS2020030206).

## Conflict of Interest

The authors declare that the research was conducted in the absence of any commercial or financial relationships that could be construed as a potential conflict of interest.

## Publisher’s Note

All claims expressed in this article are solely those of the authors and do not necessarily represent those of their affiliated organizations, or those of the publisher, the editors and the reviewers. Any product that may be evaluated in this article, or claim that may be made by its manufacturer, is not guaranteed or endorsed by the publisher.
